# Geopolymer Materials for Additive Manufacturing: Chemical Stability, Leaching Behaviour, and Radiological Safety

**DOI:** 10.3390/ma18214886

**Published:** 2025-10-24

**Authors:** Bahar Gharehpapagh, Meike Denker, Szymon Gadek, Richard Gruhn, Thomas Grab, Kinga Korniejenko, Henning Zeidler

**Affiliations:** 1 Institute for Machine Elements, Engineering Design and Manufacturing, Technische Universität Bergakademie Freiberg, 09599 Freiberg, Germanyhenning.zeidler@imkf.tu-freiberg.de (H.Z.); 2 Faculty of Materials Engineering and Physics, Cracow University of Technology, 31-155 Cracow, Poland; szymon.gadek@pk.edu.pl (S.G.); kinga.korniejenko@pk.edu.pl (K.K.); 3 Scientific Diving Center, Technische Universität Bergakademie Freiberg, 09599 Freiberg, Germany; richard.gruhn@ttd.tu-freiberg.de (R.G.); thomas.grab@ttd.tu-freiberg.de (T.G.)

**Keywords:** geopolymers, additive manufacturing, environmental compatibility

## Abstract

Geopolymers are inorganic aluminosilicate binders formed by alkali activation of reactive powders, offering a sustainable, low-carbon alternative to Portland cement. Their rapid setting and chemical durability make them well-suited for additive manufacturing (AM) in demanding environments, including underwater construction, where chemical stability is essential for both structural integrity and environmental safety. This study evaluates two metakaolin-based formulations designed for underwater extrusion, differing in activator chemistry and rheology control. Standardized leaching tests revealed alkaline but stable leachates with strong immobilization of most ions; major anions and total dissolved solids remained within regulatory thresholds. Limited exceedances were observed—soluble organic carbon in the NaOH-activated mix and arsenic/selenium in the waterglass–sand system—highlighting specific areas for mix improvement rather than fundamental limitations of the material. Complementary radioactivity screening confirmed activity concentration indices well below the regulatory limit, with measured radionuclide activities falling comfortably within exemption ranges. Together, the leaching and radioactivity results demonstrate that both formulations provide robust matrix integrity and environmental compatibility, while highlighting clear opportunities for mix design improvements to further minimize ecological risks.

## 1. Introduction

Geopolymers, formed by alkali activation of aluminosilicate-rich precursors such as metakaolin (MT) or fly ash (FA), offer a low-carbon and chemically durable alternative to Portland cement. Unlike Portland cement, their synthesis involves polycondensation reactions rather than hydration, resulting in a significantly lower carbon footprint and enhanced chemical durability. These materials offer rapid setting, thermal stability, and resistance to chemical attacks, making them attractive for applications in aggressive or specialized environments [1,2,3].

In recent years, the applicability of geopolymers has been increasingly explored within the context of additive manufacturing (AM), especially extrusion-based 3D printing [1,4]. Their adjustable rheology, early strength gain, and compatibility with thixotropic modifiers allow them to meet the processing requirements of layer-by-layer deposition. This makes them suitable prospects for automated construction, structural repair, and prefabricated components [5,6].

Geopolymers have also gained attention for use in submerged or underwater environments, where conventional concrete systems often struggle with washout, delayed setting, and reduced strength development [7]. Their intrinsic low permeability and high chemical stability offer advantages in marine construction, hydraulic structures, offshore platforms, artificial reefs, and environmental barriers [8,9]. Studies have demonstrated that geopolymer concretes can outperform traditional cementitious systems in sulphate-rich or chloride-laden waters [10] and for general durability and leaching concerns [11].

Despite this potential, the application of geopolymers in submerged 3D printing remains underexplored, particularly in terms of balancing printability with environmental safety. Achieving high shape retention and green strength while minimizing ion leaching requires carefully designed mixtures that are tailored for both rheological and chemical performance. Key factors influencing these properties include the choice of activator, solid-to-liquid ratio, filler content, and functional additives such as biopolymers and fibres [12,13,14].

The activator type and mix composition play critical roles in controlling viscosity, buildability, and setting behaviour in extrusion-based printing. The importance of optimizing pumpability, open time, and yield stress to ensure field-scale viability is emphasized in the work of Reza and Zhang [1]. Sodium hydroxide and commercial sodium silicate (waterglass) are among the most widely used activators. While NaOH accelerates precursor dissolution and supports early strength gain [10,11], it can pose handling and leaching challenges [2]. Waterglass-based systems offer a more stable reaction pathway and better compatibility with thickeners and fillers [2,3]. Silica, graphite, and cellulose improve workability and matrix densification, while biopolymers and fibres enhance shape stability and crack resistance during early stages [5,15].

Environmental considerations are especially important for geopolymers that are used in marine or civil infrastructure. Leaching behaviour serves as a key indicator of chemical stability and ecological safety, while radiological screening ensures compliance with regulatory standards for long-term applications. Geopolymer matrices are known for their ability to immobilize heavy metals due to their dense aluminosilicate networks and chemically bonded gel phases [16,17]. Fly ash-based geopolymers have shown strong resistance to leaching of toxic metals like Pb, Cd, and Cr, with immobilization rates exceeding 93.6%, even under aggressive exposure conditions [18]. This performance results from combined mechanisms of gelation, physical encapsulation, and ion exchange. Ren et al. [18] further demonstrated that increasing the CaO content enhanced both strength and heavy metal retention. Bazan et al. [19] reported low overall leachability in waste-derived geopolymers, although some formulations showed elevated arsenic and chromium levels. Additionally, recent studies confirmed the eco-suitability of 3D-printed geopolymer coral structures and insulating foams, which show low ion release and good durability in marine environments [17,19]. These findings reinforce the potential of well-designed geopolymers as sustainable, durable alternatives to Portland cement for underwater applications.

Previous works by Denker et al. [20] and Oliwa et al. [21] demonstrated the mechanical robustness and underwater durability of MT-based geopolymer systems. Denker et al. [20] reported compressive strengths of approximately 50 MPa for cast, 20 MPa for 3D-printed (air-cured), and 8–15 MPa for underwater-printed specimens, while Oliwa et al. [21] confirmed high mechanical stability and low water absorption for similar formulations exposed to aquatic environments. Building upon these findings, the present study focuses on complementary environmental validation through leaching and radioactivity assessments.

This work advances the state of the art by providing the first side-by-side environmental assessment of two MT-based geopolymer formulations that are specifically tailored for underwater extrusion. One employs a NaOH-based activator system, while the other uses commercial sodium silicate (Baucis) combined with sand and cellulose. To evaluate their environmental compatibility, standardized leaching tests were conducted on 3D-printed specimens. Here, we directly compare (i) the standardized leachability of major ions and regulated elements, and (ii) radiological safety based on activity concentration indices (I_ac_). This study closes the gap between the previously established printability and mechanical performance and the required environmental compliance for submerged use, delivering a complete, practice-oriented validation of MT-based printable geopolymers for underwater applications. The findings aim to guide environmentally responsible mix design for submerged AM with geopolymers and emphasize the potential of 3D-printed formulations to meet the chemical stability requirements of long-term underwater applications.

## 2. Materials and Methods

Geopolymers have been widely studied as sustainable alternatives to Portland cement due to their lower carbon footprint, superior chemical durability, and competitive mechanical properties. Their performance depends strongly on the precursor material, which influences reactivity, setting behaviour, workability, and long-term durability. Table 1 categorizes common geopolymer systems based on their main precursors, such as MT, FA, slag, red mud, copper slag, and rice husk ash, along with typical applications and references from the literature.

Among these systems, MT-based geopolymers stand out for their controlled chemistry, high purity, and consistent performance. They form a dense N-A-S-H gel network that provides excellent mechanical strength, low shrinkage, and resistance to thermal and chemical degradation [17,22,24]. Compared to Ca-rich systems (e.g., slag-based), MT-based geopolymers are less prone to sulphate attack and ionic diffusion, making them better suited for submerged or marine applications [16,18].

While fly ash and slag-based geopolymers are widely adopted for their workability and early strength, they often exhibit compositional variability and potential durability concerns in aggressive environments [27,38]. Waste-derived binders such as red mud and copper slag support circular economy goals but require strict control of heavy metal leaching to meet environmental standards [19,30].

Based on these factors, this study employs MT as the primary precursor for geopolymer mixtures developed for extrusion-based AM. MT enables the formulation of binders with reliable chemical stability, adjustable rheology, and high structural integrity, which are essential for 3D printing in marine environments. The subsequent sections explore how activator type, filler content, and functional additives affect printability, durability, and environmental performance.

### 2.1. Component Functions

The developed geopolymer mixtures are formulated with an emphasis on sustainability, performance, and adaptability for underwater or marine environments. Both formulations are based on an MT-rich aluminosilicate matrix and incorporate functional additives and recycled materials to enhance workability, strength, and durability. Two distinct formulations are employed: *Mixture I*, activated using a concentrated sodium hydroxide solution, and *Mixture II*, activated using a commercial waterglass solution (Baucis). The roles of individual components in each mixture are described below.

**MT:** MT serves as the primary aluminosilicate precursor for geopolymerization. Produced through the calcination of kaolinite clay, it provides a highly reactive source of silica and alumina while being significantly less energy-intensive than Portland cement. Its use supports low-carbon construction and contributes to rapid hardening, early strength development, and long-term chemical durability.

**Alkaline Activators:** Alkaline activators initiate the dissolution of the amorphous aluminosilicate phases in MT, enabling the formation of a geopolymeric gel network.

- *Mixture I* utilizes a highly concentrated sodium hydroxide solution (16 M NaOH), providing intense alkalinity that promotes rapid precursor dissolution, fast setting, and early strength development. This makes it suitable for time-sensitive or rapid buildup applications where early load-bearing is critical.
- *Mixture II* incorporates a commercial sodium silicate solution (Baucis waterglass; Ceske Lupkove Zavody, Nove Straseci, Czech Republic), offering a more balanced and controlled activation mechanism. The presence of soluble silica moderates reaction kinetics, enhances workability, and improves compatibility with thixotropic additives such as cellulose and sand, which is key for applications requiring high shape retention and delayed setting.

**Silica:** Reactive silica, such as fumed silica, silica fume, or recycled glass powder, enhances the Si content of the system and supports the formation of a denser aluminosilicate network. It improves compressive strength, reduces porosity, and enhances long-term durability.

**Graphite:** Graphite functions as a fine, inert filler that improves paste flow, mechanical cohesion, and thermal stability. Its hydrophobic and chemically stable nature enhances dimensional stability under humid or submerged conditions. However, since carbon-based materials are generally inert to both water and the alkaline components of the geopolymer matrix, excessive graphite addition could reduce chemical interactions during geopolymerization and lead to local discontinuities within the matrix, potentially affecting density and strength. When sourced from recycled battery or industrial waste streams, graphite also contributes to circular material use.

**Carbon Fibres:** Short carbon fibres (approximately 3 mm in length) act as micro-reinforcements to control early-age shrinkage and enhance tensile and flexural strength. These fibres improve crack resistance and green strength. Recycled carbon fibres from composite waste further increase the environmental benefit.

**Alginate:** Alginate, a natural biopolymer derived from seaweed, improves viscosity, cohesion, and shape retention. It enhances printability in extrusion-based or form-free applications. As a renewable and biodegradable additive, it supports the ecological profile of the formulation.

**Anhydrite:** Anhydrite (CaSO_4_), a calcium-rich additive derived from gypsum waste, acts as a mild set accelerator. It helps to promote the formation of secondary calcium–silicate–hydrate (C–S–H) phases, especially in humid or submerged curing environments. Its presence improves early-age strength and refines pore structure.

**Cellulose (*Mixture II* only):** Cellulose serves as a thickener and water-retention agent. It enhances paste stability under low water-to-solid ratios, reduces bleeding, and improves printability. Its compatibility with waterglass-based systems strengthens its role as a thixotropic modifier.

**Sand (<1 mm, *Mixture II* only):** Fine quartz sand functions as a coarse filler to reduce shrinkage and improve dimensional stability. It enhances mechanical interlocking and adjusts rheological properties, ensuring the structural integrity of fresh mixtures during shaping and curing.

The choice of alkaline activator critically affects rheology, setting behaviour, printability, and environmental performance, especially in underwater construction [7,17]. *Mixture I*, based on 16M NaOH, enables rapid precursor dissolution and early strength gain, ideal for fast-paced or early-loading scenarios [18]. However, its high alkalinity increases risks of shrinkage, efflorescence, and handling difficulty under uncontrolled curing conditions [2]. In contrast, *Mixture II* uses Baucis waterglass, which supplies both sodium and soluble silica, leading to more controlled geopolymerization, improved workability, and lower environmental impact [3,19]. These qualities make it suitable for AM, casting, and underwater placement, where delayed setting and shape retention are required [5,17].

In conclusion, the synergistic combination of these components enables the development of geopolymer mixtures that are both high-performing and environmentally responsible. Each ingredient plays a distinct role in tuning the mixture’s rheological, mechanical, and durability characteristics, while collectively advancing the goals of material circularity, reduced carbon footprint, and enhanced suitability for marine applications.

### 2.2. Mixture Composition

Two distinct geopolymer mixtures were developed and evaluated for suitability in underwater AM. Both are normalized to MT content and formulated to balance early strength, printability, and durability. The key distinction between the two lies in the choice of alkaline activator and the incorporation of rheology-modifying additives and fillers. The component ratios for both mixtures, normalized to an MT baseline of 1.00, are summarized in Table 2.

- *Mixture I* is activated using a concentrated sodium hydroxide solution (16 M NaOH) in combination with waterglass. It contains graphite and other fine-scale additives to enhance matrix cohesion and early mechanical performance.
- *Mixture II* employs a commercial sodium silicate solution (Baucis) as the sole activator. It includes additional rheology modifiers, cellulose, and sand to improve shape retention, stability, and workability during extrusion. The anhydrite content is also slightly increased to compensate for delayed setting behaviour.

As shown in Table 2, the most significant formulation differences lie in the use of coarse filler and rheology modifiers. *Mixture II* incorporates a substantial amount of fine sand (1.00 by weight), which is absent in *Mixture I*, effectively reducing the relative binder content. Graphite content is reduced from 0.10 in *Mixture I* to 0.04 in *Mixture II*, reflecting the shift toward a denser, more granular matrix in the latter. Additionally, *Mixture II* includes cellulose (0.01) to support thixotropic behaviour and a higher dosage of anhydrite (0.01 compared to 0.005) to compensate for the milder reactivity of the Baucis activator. These changes result in a more stable and shape-retaining paste in *Mixture II*, which is better suited for underwater extrusion, whereas *Mixture I* prioritizes early strength and cohesion through a finer particle system and more aggressive activation.

Although both mixtures are normalized to MT for compositional comparison, the actual MT content differs significantly when the mixtures are prepared at equal total batch weights. This is primarily due to the high sand content and additional modifiers present in *Mixture II*. As a result, the effective mass fraction of MT in *Mixture II* is nearly half that of *Mixture I*. This discrepancy is critical when interpreting strength and leaching results, as a lower binder content may influence matrix density, reaction extent, and long-term durability. Therefore, the absolute MT content should be taken into account when comparing the performance of the two formulations on a mass-equivalent basis.

### 2.3. 3D Printing Process

The geopolymer mixtures were printed using a WASP 40100 LDM equipped with a manual feeding system. The extrusion assembly featured a funnel-shaped metal tank and a gravity-assisted, screw-driven mechanism. Unlike pneumatic systems, no air pressure was applied; instead, a motorized auger enabled precise, low-pressure extrusion of highly viscous pastes.

**Figure 1:**
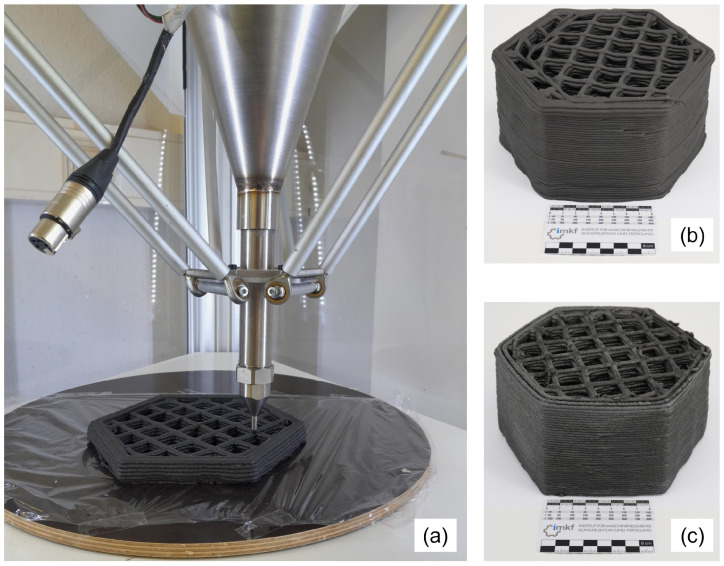
(**a**) Paste-extrusion 3D printing in progress. Completed printed parts with (**b**) *Mixture I* and (**c**) *Mixture II*.

Due to the presence of sand and 3 mm carbon fibres in the mixture, a 4 mm nozzle diameter was selected as optimal for extrusion. Given the chemical reactivity of the geopolymer with steel, a metal tank was employed as an alternative; plastic tanks are also considered suitable. The paste was manually loaded to ensure uniform compaction within the tank, thereby promoting consistent extrusion. All printing operations were conducted at room temperature, without external heating or vibration, to reflect conditions relevant to underwater or field-scale AM. The key printing parameters used in this study are summarized in Table 3, and the printing process and the resulting geometries for both mixtures are illustrated in Figure 1.

A pronounced difference in rheological behaviour was observed between the two mixtures during the printing process. *Mixture I*, activated with 16 M NaOH, exhibited high viscosity and stickiness, which reduced its workability during manual loading and extrusion. However, it provided excellent extrusion uniformity and strand cohesion, resulting in stable and well-defined layer deposition. In contrast, *Mixture II*, formulated with the commercial waterglass solution (Baucis), demonstrated lower viscosity and improved workability, enabling smoother flow through the nozzle. This facilitated easier handling but came at the expense of slightly reduced strand definition, requiring more precise control during printing. These contrasting behaviours highlight the critical role of activator chemistry in governing the flow characteristics and buildability of geopolymer pastes for extrusion-based fabrication.

## 3. Results and Discussion

### 3.1. Leaching Behaviour

Leaching tests are a widely accepted method for evaluating the chemical stability and environmental compatibility of geopolymeric materials. In this study, leachability tests were commissioned for all analyzed mixtures and conducted by AP Geotechnika Porsze, Kapica Sp. k. (Poland) in accordance with PN-EN 12457-2:2006 standard [39,40]. Major and trace metal concentrations (As, Ba, Cd, Cr, Cu, Hg, Mo, Ni, Pb, Sb, Se, Zn) in the leachate were determined using inductively coupled plasma optical emission spectroscopy (ICP-OES) following PN-EN ISO 11885:2009. Dissolved organic carbon (DOC) was measured using a TOC/DOC analyser, while anions (chlorides, fluorides, sulphates), hexavalent chromium, total dissolved solids (TDSs), and pH were determined using standard water and wastewater analysis methods.

An increase in ion concentration in the leachate, particularly of alkalis and heavy metals, may indicate incomplete geopolymerization, poor matrix integrity, or excess unreacted activators. Conversely, low leaching values suggest a chemically stable and well-polymerized matrix. By comparing the performance of the two mixtures under identical exposure conditions, insights were obtained regarding matrix immobilization efficiency, the influence of activator systems, and the compatibility of additives such as sand, cellulose, and graphite, factors that are crucial for underwater and environmentally sensitive applications. The leaching results are summarized in Table 4, including the values for soluble organic carbon (SOC) and total dissolved solids (TDSs).

**Figure 2:**
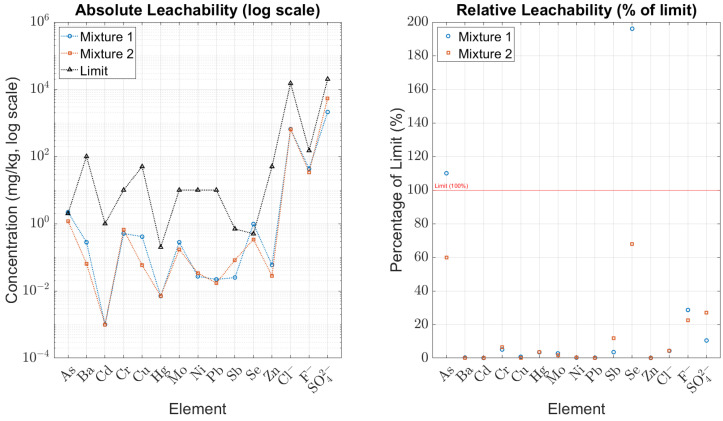
Leachability of heavy metals and anions of *Mixture I* and *Mixture II*. (**Left**) Absolute concentrations (log scale); (**Right**) relative to inert waste limits (percentage).

**Figure 3:**
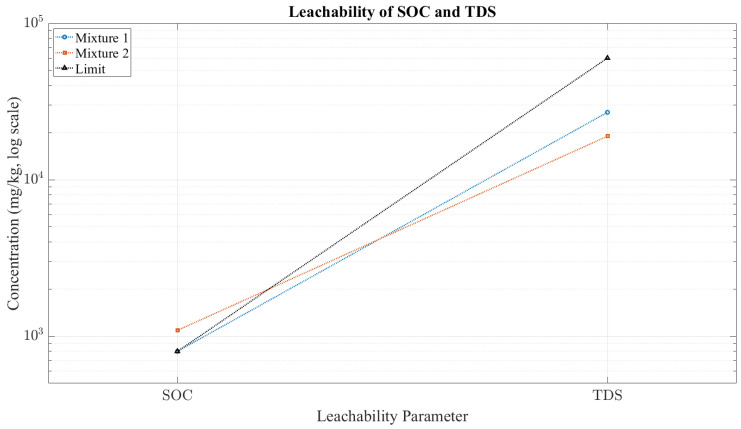
Leachability of soluble organic carbon (SOC) and total dissolved solids (TDSs) compared to inert waste limits.

As shown in Figure 2, most elements for both mixtures remain well below the regulatory limits. Exceptions include selenium and arsenic in *Mixture I*, which exceed their respective thresholds. In Figure 3, *Mixture II* slightly exceeds the SOC limit (1090 mg/kg vs. 800 mg/kg), while TDS values for both mixtures are comfortably below the permissible range. *Mixture I* meets most criteria but exceeds the thresholds for arsenic and selenium; SOC remains within the acceptable limit. *Mixture II* marginally exceeds the SOC limit but satisfies all other regulatory criteria. Both mixtures demonstrate strong immobilization of chloride, fluoride, and sulphate ions, confirming the integrity of the geopolymer matrix.

The differences observed can be linked to their compositions. *Mixture I*, activated with 16 M NaOH, contains a higher effective proportion of MT, since it is not diluted with sand. This composition promotes higher alkalinity and greater ionic mobility, which is consistent with its elevated arsenic and selenium release. In contrast, *Mixture II*, which incorporates Baucis waterglass together with a substantial sand fraction, has a lower relative MT content. This dilution effect reduces heavy metal mobility but is associated with a slight increase in SOC, likely influenced by the presence of cellulose and other organic modifiers. Both mixtures exhibit generally favourable leaching behaviour. *Mixture I* requires caution owing to its release of arsenic and selenium, while *Mixture II* is slightly non-compliant due to elevated SOC levels. Overall, the results support their potential for low-impact applications, provided that appropriate waste management protocols are followed.

### 3.2. Radioactivity Behaviour

To complement the leaching assessment, radioactivity screening was conducted on the MT-based geopolymer mixtures to evaluate their compliance with radiological safety standards for construction and A materials. The activity concentrations of ^226^Ra, ^232^Th, and ^40^K were measured, and the activity concentration index (I_ac_) was calculated following the European Commission’s Radiation Protection 112 recommendations [41].

Table 5 presents the results for both geopolymer formulations. *Mixture II* (Baucis + Sand) exhibited a notably higher ^40^K activity (1192 Bq/kg), which is attributed to the high potassium content in the waterglass activator and the natural sand fraction. Similar results were observed by Salazar et al. [42], who reported that K-bearing precursors and activators significantly increased ^40^K activity in fly ash geopolymers. In contrast, *Mixture I* (Metakaolin 1.5, NaOH 16 M) showed slightly elevated ^226^Ra and ^232^Th activities, consistent with previous findings that trace uranium and thorium occur naturally in kaolinite-derived MT clays [43,44].

Despite these compositional differences, the calculated I_ac_ values of 0.50 and 0.72 remain well below the EU regulatory limit of 1.0 for building materials, confirming the radiological safety of both mixtures. These values are comparable to the activity indices reported for other geopolymer systems—ranging from 0.3 to 0.8 for fly ash- and MT-based matrices—indicating that the studied formulations exhibit typical or lower natural radioactivity than conventional cementitious binders [42,45].

From an application perspective, these results are particularly encouraging for AM of functional or structural components that are intended for aquatic or environmentally sensitive environments. The low activity levels indicate negligible long-term environmental or occupational risks. Moreover, the aluminosilicate network of geopolymers has been shown to immobilize radionuclides effectively through chemical bonding and physical encapsulation mechanisms [43,44]. Houhou et al. [43] demonstrated that geopolymer matrices maintain high stability under irradiation and thermal exposure, with radionuclide leach rates being reduced by over 95% compared to Portland cement, while Phillip et al. [44] highlighted their long-term radiological and chemical durability during waste immobilization.

Overall, the current results, together with literature evidence on geopolymer stability and radionuclide retention, confirm that both MT-based formulations are radiologically safe. Combined with their favourable leaching performance, these findings underscore the environmental compatibility and suitability of the studied geopolymers for sustainable AM in submerged or ecologically sensitive environments.

## 4. Conclusions and Future Perspectives

This study evaluated the chemical stability and environmental performance of two MT-based geopolymer mixtures developed for extrusion-based AM in submerged conditions. Standardized leaching tests showed that both mixtures effectively immobilize most ions, with only limited exceedances: arsenic/selenium in the NaOH-activated system (*Mixture I*) and SOC in the waterglass-sand system (*Mixture II*). Radioactivity screening further confirmed that activity concentration indices remained well below the regulatory threshold, demonstrating radiological safety.

Overall, these results indicate that both formulations provide a chemically stable and environmentally compatible matrix that is suitable for underwater applications. The observed exceedances highlight targeted areas for mix optimization but do not reflect fundamental material limitations. Future improvements may focus on fine-tuning activator chemistry and additive selection to further reduce ion release, thereby enhancing the long-term ecological compatibility of 3D-printed geopolymer structures in marine and hydraulic environments.

Future research should focus on refining activator compositions and incorporating tailored additives to further reduce soluble organic carbon and trace element release. Although sodium ion (Na^+^) concentrations were not measured in this study, their quantification will be an important part of future work to better assess alkali leaching behaviour and confirm the long-term chemical stability of the developed geopolymer systems. Moreover, long-term durability studies under realistic marine exposure conditions, including cyclic loading and biofouling, will also be essential to confirm field applicability. Finally, scaling the process to larger 3D-printed structures and assessing their mechanical and environmental performance in situ will provide critical insights for real-world deployment.

## Figures and Tables

**Table 1:** Categorization of Geopolymers based on Main Precursor and their Suitability for Submarine Applications.

Geopolymer Type	Properties	Applications	References
Metakaolin	High amorphous Si/Al content; rapid hardening and high early strength; excellent chemical durability; low shrinkage; predictable setting and consistent workability.	Precast elements, structural repair, chemically resistant underwater binders.	[22,23,24]
Fly Ash	Class F: low-Ca, forms N-A-S-H gel; Class C: Ca improves early strength but may reduce durability if uncured; variable fresh-state workability; often requires thermal or ambient curing.	Eco-concretes, structural components; limited underwater use without additives.	[25,26]
Slag	High CaO content; forms C-A-S-H gel; improves early strength at ambient temperatures; durable in mild seawater and under freeze–thaw cycles; rapid setting and slump loss unless modified with admixtures; enhances mechanical and bond strength; good abrasion resistance.	Marine structures, precast marine components, sewer/infrastructure linings; promising for underwater repairs.	[27,28]
Natural Pozzolan	Moderate reactivity; requires NaOH/KOH activation or blending; slower setting and lower early workability; sustainable but with moderate strength and durability.	Rural, earthen, low-carbon construction.	[29]
Red Mud	High Fe_2_O_3_/Al_2_O_3_ ratio; requires strong alkali activation or slag blending; effective for heavy metal immobilization; irregular workability and setting behaviour; durability is formulation-dependent.	Waste stabilization, eco-bricks, confined damping blocks.	[30,31]
Copper Slag	High Fe/Si, low Al content; low reactivity; enhances matrix density and abrasion resistance; ambient-cured matrices are compact but have lower strength; requires blending or chemical activation; heavy metal leaching is manageable.	Industrial flooring, abrasion-resistant tiles; cautious use in submerged conditions.	[32,33,34,35]
Rice Husk Ash	High amorphous silica content; refines pore structure and increases matrix density; significantly improves compressive strength in optimized blends; reduces workability; accelerates setting; lightweight; durable when combined with ground granulated blast furnace slag or bauxite.	Lightweight marine composites, blended alkali-activated binders.	[36,37]

**Table 2:** Component ratios of the two geopolymer mixtures (normalized to MT = 1.00).

Component	Mixture I (NaOH 16 M)	Mixture II (Baucis + Sand)
Metakaolin	1.00	1.00
Waterglass	0.90 (NaOH 16 M)	0.90 (Baucis)
Silica	0.10	0.10
Graphite	0.10	0.04
Carbon fibers	0.01	0.01
Alginate	0.01	0.01
Anhydrite	0.005	0.01
Cellulose	–	0.01
Sand (<1 mm)	–	1.00

**Table 3:** Three-dimensional printing parameters used for the geopolymer mixtures.

Parameter	Value
Printer model	WASP FOR 40100 CLAY
Feeding system	Manual screw-based (without air pressure)
Tank shape	Funnel (metal)
Nozzle diameter	4 mm
Layer height	2 mm
Print speed	80 mm/s
Infill pattern	Grid (rectilinear)
Infill density	25%
Print temperature	Ambient (no heating)

**Table 4:** Leachability results compared to inert waste limits [40].

Characteristic	Mixture I (mg/kg)	Mixture II (mg/kg)	Limit (mg/kg)
Arsenic (As)	**2.2**	1.2	2
Barium (Ba)	0.280	0.065	100
Cadmium (Cd)	<0.0010	0.0010	1
Total Chromium (Cr)	0.510	0.660	10
Chromium (Cr VI)	0.32	0.31	–
Copper (Cu)	0.410	0.058	50
Mercury (Hg)	0.0070	<0.0070	0.2
Molybdenum (Mo)	0.280	0.170	10
Nickel (Ni)	0.027	0.034	10
Lead (Pb)	0.022	0.017	10
Antimony (Sb)	0.025	0.083	0.7
Selenium (Se)	**0.980**	0.340	0.5
Zinc (Zn)	0.059	0.028	50
Chlorides	650	650	15,000
Fluorides	43	34	150
Sulphates	2100	5400	20,000
SOC	800	**1090**	800
TDS	27,000	19,000	60,000
pH [-]	11	11	–

**Table 5:** Activity concentrations of natural radionuclides and activity concentration index (I_ac_) for geopolymer mixtures.

Mixture	^226^Ra (Bq/kg)	^232^Th (Bq/kg)	^40^K (Bq/kg)	I_ac_
Mixture I	56.06 ± 6.22	53.79 ± 4.19	147.55 ± 2.63	0.50 ± 0.04
Mixture II	42.36 ± 4.93	37.95 ± 3.33	1192.07 ± 69.27	0.72 ± 0.05
Regulatory limit	300	200	3000	≤1

## Data Availability

The original contributions presented in this study are included in the article. Further inquiries can be directed to the corresponding author.

## References

[1] Raza M.H., Zhong R.Y. (2022). A sustainable roadmap for additive manufacturing using geopolymers in construction industry. Resour. Conserv. Recycl..

[2] Scanferla P., Gharzouni A., Texier-Mandoki N., Bourbon X., de la Plaza I.S., Rossignol S. (2025). Polycondensation reaction effect on the thermal behavior of metakaolin-based potassium geopolymers. J. Sol-Gel Sci. Technol..

[3] Alghamdi H., Neithalath N. (2019). Synthesis and characterization of 3d-printable geopolymeric foams for thermally efficient building envelope materials. Cem. Concr. Compos..

[4] Lazorenko G., Kasprzhitskii A. (2022). Geopolymer additive manufacturing: A review. Addit. Manuf..

[5] Ricciotti L., Apicella A., Perrotta V., Aversa R. (2023). Geopolymer materials for extrusion-based 3d-printing: A review. Polymers.

[6] Archez J., Texier-Mandoki N., Bourbon X., Caron J.F., Rossignol S. (2021). Shaping of geopolymer composites by 3d printing. J. Build. Eng..

[7] Li L.G., Zhang G.-H. (2024). Feasibility of underwater 3d printing: Effects of anti-washout admixtures on printability and strength of mortar. J. Build. Eng..

[8] Korniejenko K., Gądek S., Dynowski P., Tran D.H., Rudziewicz M., Pose S., Grab T. (2024). Additive manufacturing in underwater applications. Appl. Sci..

[9] Korniejenko K., Oliwa K., Gądek S., Dynowski P., Źróbek A., Lin W.-T. (2025). A review of additive manufacturing techniques in artificial reef construction: Materials, processes, and ecological impact. Appl. Sci..

[10] Dai X., Tao Y., Zhang Y., Ding L., Tittelboom K.V., Schutter G.D. (2024). Development of 3d printable alkali-activated slag-metakaolin concrete. Constr. Build. Mater..

[11] Ilcan H., Sahin O., Kul A., Ozcelikci E., Sahmaran M. (2023). Rheological property and extrudability performance assessment of construction and demolition waste-based geopolymer mortars with varied testing protocols. Cem. Concr. Compos..

[12] Hwalla J., Saba M., Assaad J., El-Hassan H., Kioumarsi M., Shafei B. (2025). Performance of metakaolin-based alkali-activated mortar for underwater placement. The 1st International Conference on Net-Zero Built Environment.

[13] Ly O., Yoris-Nobile A.I., Sebaibi N., Blanco-Fernandez E., Boutouil M., Castro-Fresno D., Hall A.E., Herbert R.J.H., Deboucha W., Reis B. (2021). Optimisation of 3d printed concrete for artificial reefs: Biofouling and mechanical analysis. Constr. Build. Mater..

[14] Yoris-Nobile A.I., Slebi-Acevedo C.J., Lizasoain-Arteaga E., Indacoechea-Vega I., Blanco-Fernandez E., Castro-Fresno D., Alonso-Estebanez A., Alonso-Cañon S., Real-Gutierrez C., Boukhelf F. (2023). Artificial reefs built by 3d printing: Systematisation in the design, material selection and fabrication. Constr. Build. Mater..

[15] Chen K., Liu Q., Chen B., Zhang S., Ferrara L., Li W. (2024). Effect of raw materials on the performance of 3d printing geopolymer: A review. J. Build. Eng..

[16] Wang S., Yu L., Xu L., Wu K., Yang Z. (2021). The failure mechanisms of precast geopolymer after water immersion. Materials.

[17] Martins J.R., Novais R.M., Hotza D., Labrincha J.A., Senff L. (2025). Waste-derived geopolymers for artificial coral development by 3d printing. J. Sustain. Metall..

[18] Ren X., Wang F., He X., Hu X. (2024). Resistance and durability of fly ash based geopolymer for heavy metal immobilization: Properties and mechanism. RSC Adv..

[19] Bazan P., Figiela B., Kozub B., Łach M., Mróz K., Melnychuk M., Korniejenko K. (2024). Geopolymer foam with low thermal conductivity based on industrial waste. Materials.

[20] Denker M., Gharehpapagh B., Gruhn R., Pose S., Korniejenko K., Grab T., Zeidler H. (2025). Compressive strength of geopolymer with recycled carbon fibres manufactured in air and in water by casting and additive manufacturing. Front. Built Environ..

[21] Oliwa K., Kozub B., Łoś K., Łoś P., Korniejenko K. (2025). Assessment of durability and degradation resistance of geopolymer composites in water environments. Materials.

[22] Duxson P., Fernández-Jiménez A., Provis J.L., Lukey G.C., Palomo A., van Deventer J.S.J. (2007). Geopolymer technology: The current state of the art. J. Mater. Sci..

[23] Onyelowe K.C., Kamchoom V., Ebid A.M., Hanandeh S., Llamuca J.L.L., Yachambay F.P.L., Palta J.L.A., Vishnupriyan M., Avudaiappan S. (2025). Optimizing the utilization of metakaolin in pre-cured geopolymer concrete using ensemble and symbolic regressions. Sci. Rep..

[24] Işıkdağ B., Yalghuz M.R. (2023). Strength development and durability of metakaolin geopolymer mortars containing pozzolans under different curing conditions. Minerals.

[25] Qaidi S., Najm H.M., Abed S.M., Ahmed H.U., Dughaishi H.A., Lawati J.A., Sabri M.M., Alkhatib F., Milad A. (2022). Fly ash-based geopolymer composites: A review of the compressive strength and microstructure analysis. Materials.

[26] Gaurav G., Kandpal S.C., Mishra D., Kotoky N. (2024). A comprehensive review on fly ash-based geopolymer: A pathway for sustainable future. J. Sustain. Cem.-Based Mater..

[27] Amer I., Abdelkhalik A., Mayhoub O.A., Kohail M. (2024). Development of sustainable slag-based geopolymer concrete using different types of chemical admixtures. Int. J. Concr. Struct. Mater..

[28] Gökçe H.S. (2024). Durability of slag-based alkali-activated materials: A critical review. J. Aust. Ceram. Soc..

[29] Hossain M.M., Karim M.R., Hossain M.K., Islam M.N., Zain M.F.M. (2015). Durability of mortar and concrete containing alkali-activated binder with pozzolans: A review. Constr. Build. Mater..

[30] Qin T., Luo H., Han R., Zhao Y., Chen L., Liu M., Gui Z., Xing J., Chen D., He B.-J. (2024). Red mud in combination with construction waste red bricks for the preparation of low-carbon binder materials: Design and material characterization. Buildings.

[31] Jiang J., Cai X., Ou X., Zhao X., Wei D., Wang S., Luo Q., Huang Y. (2025). Study on the heavy metal immobilization mechanism in the alkali-activated red mud-ground granulated blast furnace slag-based geopolymer. Matéria.

[32] You N., Chen Z., Gao Z., Song X. (2024). The effect of copper slag as a precursor on the mechanical properties, shrinkage and pore structure of alkali-activated slag-copper slag mortar. J. Build. Eng..

[33] Jin Q., Chen L. (2022). A review of the influence of copper slag on the properties of cement-based materials. Materials.

[34] Maqbool Q., Mobili A., Blasi E., Sabbatini S., Ruello M.L., Tittarelli F. (2024). Sustainable alkali-activated mortars for the immobilization of heavy metals from copper mine tailings. ACS Sustain. Resour. Manag..

[35] Seeni B.S., Maheswaran C., Nakarajan A. (2025). Effect of copper slag addition on the properties of ambient cured alkali-activated pervious concrete. Road Mater. Pavement Des..

[36] Swaminathen A.N., Kumar C.V., Ravi S.R., Debnath S. (2021). Evaluation of strength and durability assessment for the impact of rice husk ash and metakaolin at high performance concrete mixes. Mater. Today Proc..

[37] Moulick K.K., Shiuly A., Bhattacharjya S., Sau D. (2024). Optimization of rice husk ash-based alkali activated composites (aac) blended with bauxite and ggbs for sustainable building materials. Discov. Civ. Eng..

[38] Becher A.F., Zeidler H., Gądek S., Korniejenko K. (2025). Shaping and characterization of additively manufactured geopolymer materials for underwater applications. Appl. Sci..

[39] (2006). Characterisation of Waste—Leaching—Compliance Test for Leaching of Granular Waste Materials and Sludges—Part 2: One Stage Batch Test at a Liquid to Solid Ratio of 10 l/kg for Materials with Particle Size Below 4 mm (Without or With Size Reduction).

[40] (2015). Rozporządzenie Ministra Gospodarki z Dnia 16 Lipca 2015 r. w Sprawie Dopuszczania Odpadów do skłAdowania na skłAdowiskach. Dziennik Ustaw 2015, poz. 1277. https://isap.sejm.gov.pl/isap.nsf/download.xsp/WDU20150001277/O/D20151277.pdf.

[41] European Commission, Radiation Protection Unit (1999). Radiological Protection Principles Applicable to the Natural Radioactivity of Building Materials.

[42] Salazar P.A., Fernández C.L., Luna-Galiano Y., Sánchez R.V., Fernández-Pereira C. (2022). Physical, mechanical and radiological characteristics of a fly ash geopolymer incorporating titanium dioxide waste as passive fire insulating material in steel structures. Materials.

[43] Houhou M., Leklou N., Ranaivomanana H., Penot J.D., de Barros S. (2025). Geopolymers in nuclear waste storage and immobilization: Mechanisms, applications, and challenges. Discov. Appl. Sci..

[44] Phillip E., Choo T.F., Khairuddin N.W.A., Abdel Rahman R.O. (2023). On the sustainable utilization of geopolymers for safe management of radioactive waste: A review. Sustainability.

[45] Pławecka K., Bazan P., Lin W., Korniejenko K., Sitarz M., Nykiel M. (2022). Development of geopolymers based on fly ashes from different combustion processes. Polymers.

